# Elucidating the potential of crude cell extracts for producing pyruvate from glucose

**DOI:** 10.1093/synbio/ysy006

**Published:** 2018-05-14

**Authors:** David C Garcia, Benjamin P Mohr, Jakob T Dovgan, Gregory B Hurst, Robert F Standaert, Mitchel J Doktycz

**Affiliations:** 1 Bredesen Center for Interdisciplinary Research, University of Tennessee, Knoxville, TN, USA; 2 Biosciences Division, Oak Ridge National Laboratory, Oak Ridge, TN, USA; 3 Chemical Sciences Division, Oak Ridge National Laboratory, Oak Ridge, TN, USA

**Keywords:** crude extract, proteomics, glycolysis, metabolic engineering, pyruvate

## Abstract

Living systems possess a rich biochemistry that can be harnessed through metabolic engineering to produce valuable therapeutics, fuels and fine chemicals. In spite of the tools created for this purpose, many organisms tend to be recalcitrant to modification or difficult to optimize. Crude cellular extracts, made by lysis of cells, possess much of the same biochemical capability, but in an easier to manipulate context. Metabolic engineering in crude extracts, or cell-free metabolic engineering, can harness these capabilities to feed heterologous pathways for metabolite production and serve as a platform for pathway optimization. However, the inherent biochemical potential of a crude extract remains ill-defined, and consequently, the use of such extracts can result in inefficient processes and unintended side products. Herein, we show that changes in cell growth conditions lead to changes in the enzymatic activity of crude cell extracts and result in different abilities to produce the central biochemical precursor pyruvate when fed glucose. Proteomic analyses coupled with metabolite measurements uncover the diverse biochemical capabilities of these different crude extract preparations and provide a framework for how analytical measurements can be used to inform and improve crude extract performance. Such informed developments can allow enrichment of crude extracts with pathways that promote or deplete particular metabolic processes and aid in the metabolic engineering of defined products.

## 1. Introduction

Synthetic biology aims to manipulate and exploit the existing biochemical functions of livings organisms for desired purposes. Unfortunately, efforts to engineer these systems to unlock their diverse metabolic potential require developing clever methodologies to overcome aspects of the machinery that the organism uses for survival. *In vitro* synthetic biology offers an alternative way to harness an organism’s rich metabolism; it is driven by the prospect of easy to manipulate, static systems (1). Livings cells require membranes, energy and building blocks for growth and dynamic regulation of their biochemical processes. By removing the requirement to sustain life, *in vitro* systems can sidestep many of the barriers to manipulation and present an ideal system for metabolic engineering.

In their most basic form, *in vitro* systems for metabolic engineering lack the genetic material and membranes inherent to a living system. Such *in vitro*, or cell-free metabolic engineering (CFME), approaches enable the use of techniques usually reserved for chemical engineering approaches such as continuous reaction monitoring, allowing for greater control over enzymes and metabolite concentrations (2,3). Coupled with systems biology tools for flux balance analysis and elementary mode analysis, *in vitro* systems present a potent platform for bioproduction (4). The absence of a cell wall and membrane facilitates the exchange of substrate to, and product from, the system and simplifies reaction work up. Removal of the genome shuts down much of the cell’s instructional programming and eliminates the need to cope with a continually growing and changing system. This enables biosynthesis pathways to be engineered *in vitro*, minimizing carbon and energy lost to growth. Additionally, this minimizes the management of feedback regulation and allows for the production of metabolites that would be toxic to intact cells (5).

Ideally, a CFME system would contain only the components necessary to carry out the desired biochemical process. One promising approach for complex chemical conversion uses a defined set of purified enzymes. This methodology has been successfully demonstrated for hydrogen production and protein synthesis among others (6,7). While recent efforts in co-purification of full reaction cascades have reduced costs, any process utilizing bulk purified proteins remains expensive (8,9). To date, the use of purified components for CFME has resulted in long running systems capable of catalyzing reactions for several days, but with the drawback of slow catalysis rates. Novel work by Korman *et al.* (10) on the production of limonene showcases the strengths and limitations of this approach. Additionally, optimization of purified systems depends on ample information about the pathway and the involved proteins. These methods may fail to include accessory proteins which can improve pathway yield.

Crude cell extracts are finding increasing applications as alternatives to purified enzyme systems for metabolic engineering. Cell growth, followed by lysis and minimal fractionation can rapidly create robust biochemical systems for a fraction of the cost of purified enzymes. These systems contain the same enzymes and much of the same biochemistry as living systems and can serve as a proxy for the engineering of metabolite production by conventional, *in vivo* metabolic engineering. Recent work has demonstrated crude extracts as a platform for bioproduction as well, due to reduced costs of scale up and their compatibility with traditional chemical reactors (11,12). Further, early work in the optimization of bacterial cell-free protein synthesis (CFPS) systems demonstrated the ability of crude cell extracts to energize translation *in vitro* through the consumption of glucose or other glycolytic intermediates (13,14). Glucose conversion is accomplished through the 10-step enzymatic process of glycolysis starting with the phosphorylation of glucose to glucose-6-phosphate and producing ATP through a series of substrate level phosphorylations. As shown in the aforementioned works, crude *Escherichia coli* extracts can metabolize low-cost feedstocks like glucose to provide key intermediates and energy that can be drawn upon for myriad applications. The limits of the flexibility of crude extracts, granted by their inherently diverse biochemistry, remain uncertain. The proteome that enables these capabilities is only beginning to be explored and the extract preparation variables that influence this proteome require illumination (15). Proteomic analyses coupled with metabolite measurements can be used to identify and characterized the biochemical pathways capable of being supported by crude extracts.

## 2. Materials and methods

### 2.1 Cell-free extract preparation

Cell extracts were prepared from *E. coli* BL21 Star (DE3) grown at 37°C in one of three media: M9-fructose (11.1 mg l^−^^1^, CaCl_2_, 0.120 g l^−^^1^ MgSO_4_, 4.0 g l^−^^1^ fructose, 0.15 g l^−^^1^ KH_2_PO_4_, 3.39 g l^−^^1^ Na_2_HPO_4_, 0.25 g l^−^^1^ NaCl, 0.5 g l^−^^1^ NH_4_Cl); lysogeny broth (LB: 10 g l^−^^1^ tryptone, 5 g l^−^^1^ yeast extract, 10 g l^−^^1^ NaCl); or 2xYPTG (16 g l^−^^1^ tryptone, 10 g l^−^^1^ yeast extract, 5 g l^−^^1^ NaCl, 7 g l^−^^1^ KH_2_PO_4_, 3 g l^−^^1^ K_2_HPO_4_, 18 g l^−^^1^ glucose). The extracts prepared from these media are referred to as, deprived fructose (DF), LB and YT, respectively. Cell extracts were prepared by harvesting 50-ml cultures grown in baffled Erlenmeyer flasks to an OD_600_ of 1.0 for DF, 2.0 for LB or 4.0 for mid-log phase YT (YT-M). The DF cells were additionally transferred to M9 salt solution containing no fructose for 24 h before harvesting. A second-type of YT extract, YT-E, was prepared by growing cells to an OD_600_ of 2.8 and harvesting. Cells were harvested by centrifugation at 5000 ×*g* for 10 min in 50 m; volumes and washed twice with S30 buffer (14 mM magnesium acetate, 60 mM potassium glutamate, 1 mM dithiothreitol (DTT) and 10 mM Tris-acetate, pH 8.2) by resuspension and centrifugation. After the final centrifugation, pellets were weighed, flash-frozen in liquid nitrogen and stored at −80°C. For extract preparation, cells were thawed and resuspended in 0.8 ml of S30 buffer per mg of cell wet weight before sonicating using 530 joules per ml of suspension at 50% tip amplitude with ice water cooling. After sonication, the cell-slurry was centrifuged twice for 10 min at 21 100 ×*g* at 4°C, aliquoted, flash-frozen and stored at −80°C.

### 2.2 Cell-free reactions

Cell free glucose conversion reactions were carried out at 37°C for 24 h in 25 μl volumes with a final concentration 250 mM glucose, 18 mM magnesium glutamate, 15 mM ammonium glutamate, 195 mM potassium glutamate, 1 mM ATP, 150 mM Bis-Tris, 1 mM NAD^+^ and 10 mM dipotassium phosphate. Pyruvate consumption reactions were carried out using the same conditions and reagents, with the exception of glucose being replaced with 25 mM pyruvate. Growth enriched extracts were added to a final protein concentration of 4 mg ml^−1^. The reactions were quenched by the addition of an equal volume of 5% trichloroacetic acid. The supernatant after centrifugation at 11 000 ×*g* for 15 min was used for analytical measurements.

### 2.3 Analytical measurements

High-performance liquid chromatography (HPLC) was used to measure pyruvate and glucose in the cell-free reactions. An Agilent 1260 series HPLC system equipped with a diode array UV-visible detector (Agilent, Santa Clara, CA, USA) reading at 191 nm, with an Aminex HPX 87-H column (Bio-Rad, Hercules, CA, USA) was used for the quantifications. Analytes were eluted with isocratic 5 mM sulfuric acid at a flow rate of 0.55 ml min^−1^ at 35°C for 25 min.

### 2.4 Proteomics

CFME extracts were denatured with 6 M guanidinium chloride for 1 h at 60°C and allowed to cool to room temperature. Cysteines were reduced by incubation in 2 mM tris(2-carboxyethyl)phosphine hydrochloride (TCEP) for 20 min at room temperature and carboxamidomethylated by incubation in 10 mM iodoacetamide in the dark for 15 min. Samples were diluted with 5 volumes of digestion buffer (50 mM Tris-HCl, 10 mM CaCl_2_, pH 7.6), and the proteins were digested by adding trypsin at a 1:50 weight ratio (based on Bradford assay) with overnight incubation at 37°C. An additional identical amount of trypsin was then added, with an additional 4 h incubation at 37°C. Trypsin was inactivated by addition of formic acid to a final concentration of 0.1%. Tryptic peptides were obtained by centrifugation through a 10 kDa molecular weight cutoff filter (Microcon YM-10, Millipore, Billerica, MA, USA) for 20 min at 14 000 ×*g*. In total, 50 µg of tryptic digests were loaded onto a strong cation exchange resin (SCX) (Luna, Phenomenex, Torrance, CA, USA) and desalted. Digests were analyzed by two-dimensional liquid chromatography-tandem mass spectrometry (16). Briefly, peptides were eluted from SCX resin with an eleven-step gradient of aqueous ammonium acetate (50 mM to 500 mM) onto reverse-phase C18 resin (Aqua, Phenomenex, Torrance, CA, USA). Peptides were eluted from reverse-phase over two hours with a gradient from 100% solvent A (5% CH_3_CN, 0.1% formic acid in water) to 50:50 solvent A:solvent B (70% CH_3_CN, 0.1% formic acid in water). Peptides eluted from the column were introduced into the linear ion trap mass spectrometer (LTQ-XL, ThermoScientific) by nanoelectrospray. Peptide identifications were obtained from MS/MS spectra using the program Myrimatch (version 2.1.138) and compared against the *E. coli* BL21 (DE3) proteome (UP000002032), and protein identifications were assembled from peptide identifications using IDPicker, version 3.1.599 (17,18). KEGG Orthologies and Enzyme Commission numbers were assigned by BlastKOALA (19). Full tables of detected proteins, tryptic peptides and KEGG orthology assignments are deposited in Supplementary Tables S1 and S2. Descriptions of these tables and their legends are supplied in Supplementary Information.

### 2.5 Statistical analysis

Three biological replicates were used for all HPLC measurements. Error bars in figures represent ± 1*σ*. Proteome analyses were likewise performed on three biological replicates. Significant changes in protein abundance for a given pair of treatments were identified using *T*-tests (two-tailed, unpaired, equal variances) on log10-transformed normalized spectral abundance factor (NSAF) value, with Benjamini-Hochberg correction for multiple hypothesis testing. Differential abundance was determined by analysis of variance and direction of regulation by comparisons of prevalence value as previously described (15). Gene set enrichment analysis (GSEA) was performed using the tools designed by Subramanian *et al.* (20). In brief GSEA was performed using gene ontology (GO) terms and Uniprot pathway and superpathway annotations as pairwise comparisons of log10 transformed NSAF values between each set of extracts. Gene sets enriched with a false discovery rate <25% were retained.

## 3. Results

Given that the metabolic capabilities of a cell-free extract result from the active proteome, we hypothesized that changes to growth conditions prior to preparation of cell extracts would create shifts in the protein content and the resulting metabolic abilities of the crude extracts. With the goal of investigating the protein elements of crude cell extracts that influence precursor supply, pyruvate biosynthesis in crude extracts was assessed. Pyruvate is both an important compound central to carbon metabolism, linking glycolysis and Krebs cycle and a launching point for numerous biotechnological targets (21). Proteome profiles were obtained for the resulting crude extracts and validated by measuring the extracts’ ability to produce pyruvate after the addition of glucose.

The effects of four growth conditions on the protein content and metabolic ability of *E. coli* crude extracts to produce pyruvate from glucose, were assessed. Three different growth media were used: standard rich broth (LB), M9 minimal medium with fructose (DF) and extra-rich broth (2xYTPG, YT) with cells collected at mid-log phase. Cell growth in the 2xYPTG medium saturates at an OD_600_ of 8-10. Cells grown in this media were collected at both early, (OD_600_ 2.8) and mid-log phase (OD_600_ 4.0), and are referred to as YT-E and YT-M, respectively. The 2xYPTG condition, collected in early-log phase growth, is commonly used for CFPS (22). Cells collected early in log phase growth have the greatest specific growth rate, a parameter that is suggested to influence CFPS capabilities and may affect the abundance of glycolytic enzymes (23,24). These growth conditions were chosen based on variables with the potential to enrich for glycolytic enzymes and for their frequent use for bacterial growth and related experiments that employ crude cell extracts. The DF condition employed M9 medium with fructose as the carbon source and a starvation regimen, which is reported to increase expression of glycolytic enzymes (25).

### 3.1 Effects of growth conditions on proteomes of extracts

**Figure 1.**
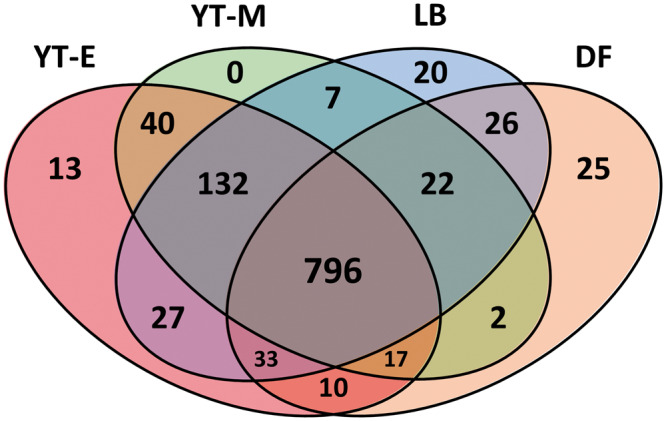
Venn diagram representing proteins found in each of the four growth conditions after filtering out low abundance proteins. There is a core set of 796 proteins present in all four conditions. The different conditions contain a small number of unique proteins, with the exception of YT-M, which may be a subset of the YT-E conditions. The cell extracts grown in rich media (YT-E, YT-M, LB) share a greater number of proteins (132) than any other subset of these conditions.

Three biological replicates of the chosen cell extracts were digested with trypsin, and tryptic peptides were analyzed using multidimensional protein identification technology (MudPIT) as previously described (16). Proteins were identified and assigned functions by matching peptides against an *E. coli* BL21 (DE3) genome that had previously been annotated by KEGG’s BlastKOALA software (19). After removal of low abundance proteins and data filtering, 1170 unique proteins were identified across all four conditions, with a core set of 796 proteins present in all four conditions. Despite overlap in media components between several of the growth conditions, there were measurable differences in the proteomes of the four resulting cell-extracts. Excluding YT-M, each growth condition contained at least 10 unique proteins (Figure 1). As the same 2xYTPG media is used for both the YT-M and YT-E conditions the conditions overlap strongly, a larger number of proteins (40) are shared and are distinct from the LB and DF conditions. Further, the rich media conditions (LB, YT-E, YT-M) share a large number of proteins (132) that are distinct from the minimal media based DF condition.

**Figure 2.**
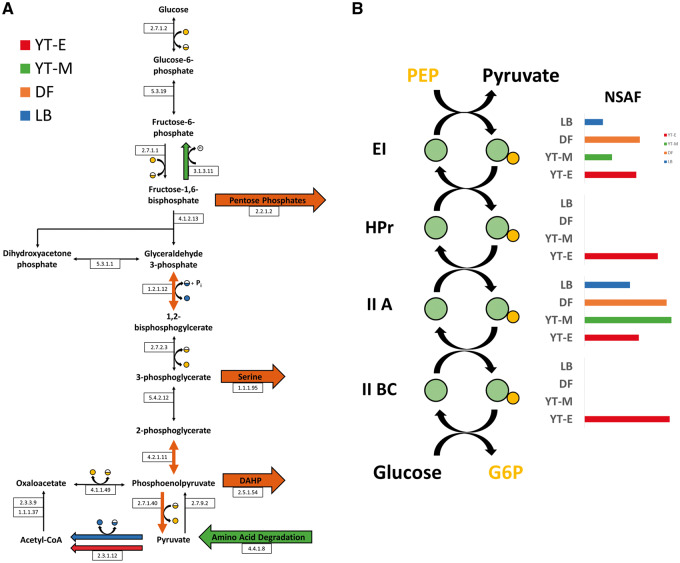
(**A**) Proteomic assessment of the potential *in vitro* metabolic fates of glycolytic intermediates. Enzymatic steps are represented by Enzyme Commission (EC) numbers. Each EC number and reaction arrow corresponds to at least one protein detected in all growth conditions. Upregulated steps are represented by red, green, orange and blue colored arrows for YT-E, YT-M, DF and LB growth conditions, respectively. Only differentially abundant sinks of glycolytic intermediates are shown. Cofactors are depicted as colored circles, full yellow circle, ATP, half yellow circle; ADP, full blue circle, NADH, half blue circle; NAD+, ‘Pi’ within the empty circle, inorganic phosphate. (**B**) Phosphoenolpyruvate transferase system reaction schematic. General PEP phosphotransferase enzyme, EI, is upregulated in both the YT-E and DF cell extracts. HPr phosphotransferase protein and glucose-specific IIA protein are only present in YT-E condition. Enrichment is represented by NSAFs.

With the goal of characterizing the proteins contributing to pyruvate production, the 10 enzymes comprising glycolysis were analyzed. In *E. coli*, four of the glycolytic enzymes exist as isoforms; 6-phosphofructokinase [Enzyme Commission (EC) number 2.7.1.11], glycerol-3-phosphate dehydrogenase (1.2.1.12), phosphoglycerate mutase (5.4.2.12) and pyruvate kinase (2.7.1.40), which may be regulated and function differently in a living cell. As the origin of these differences is beyond the scope of this work, each enzyme group is henceforth represented functionally as an EC number shared within isoforms. At least one isoform of each of the 10 glycolytic enzymes is present in all 4 conditions (Figure 2). While glycolysis is predominantly regulated through allosteric interactions, differences in abundance of the participating enzymes may also affect flux through the pathway (26). As shown in Figure 2, three enzymes within glycolysis are upregulated in the DF condition (1.2.1.12, 4.2.1.11 and 2.7.1.40).

In addition to energy, glycolysis provides the metabolic feedstocks for many important pathways, including the pentose phosphate pathway and the Krebs cycle, which in turn are the source of the carbon backbone for most primary and secondary metabolites in bacteria. The many carbon sinks leading out of glycolysis represent a significant draw away from pyruvate production. To account for the effect of these pathways, abundances of every enzyme in the BL21 (DE3) genome known to interact with an intermediate or product of glycolysis were analyzed for differences. These results are compiled in Supplementary Table S3. Figure 2 depicts the key differentially abundant enzymes in the cell extract proteomes that can act on glycolytic molecules. In particular, the pathway to the aromatic amino acid precursor shikimate was differentially represented via the pentose phosphate pathway enzyme transaldolase (2.2.1.2) and 3-deoxy-D-arabino-2-heptulosonic acid 7-phosphate (DAHP) synthetase (2.5.1.54).

The 10-enzyme glycolytic pathway from glucose to pyruvate begins with a phosphorylation that can be performed by hexokinase (2.7.1.2). This enzyme is present in all four extract conditions, but the phosphoenolpyruvate phosphotransferase system (PTS) provides an alternative entry point into glycolysis. PTS is a multi-protein phosphorylation cascade that *in vivo* results in a phosphorylated sugar moiety using PEP as an energy source. Previous work *in vitro* has demonstrated activity of the glucokinase and PEP phosphatase enzymes in crude extracts (27). PTS specificity is dictated by the non-membrane bound IIA enzyme and membrane-bound IIBC enzyme, which often will only phosphorylate a single sugar, allowing for selective import of dedicated sugars. The EI PTS protein is upregulated in both the YT-E and DF extract (Figure 2). While the DF extract condition contains the fructose/mannitol-specific IIBC protein, the YT-E extract proteome uniquely contains the glucose-specific BCII enzyme.

Analysis of individual enzymes helps to predict the flow of carbon through a cell extract. However analysis of individual abundances may fail to detect systematic differences between cell extracts. Sets of phenotypically related genes can be co-regulated, but individually fail pairwise tests of significance. GSEA was performed using GO terms and Uniprot pathway designations annotated with the genome to account for these differences (20). GSEA was performed as pairs of comparisons but some enrichments were shared amongst different comparisons and were consolidated (Table 1).

**Table 1.** Summary of gene set enrichment analysis results based on biological process and molecular function GO terms and Uniprot Superpathway annotations

Condition	Enrichment	Description	Direction of regulation	Comparison
YT-E	Carbohydrate metabolism		Down	LB, DF
YT-M	Purine metabolism		Up	YT-E
YT-M	Cofactor biosynthesis		Up	YT-E
DF	Amino-acid biosynthesis		Up	YT-E, LB
DF	Carbohydrate degradation		Up	LB
DF	GO:0000049	tRNA binding	Down	YT-M, YT-E
DF	GO:0003676	Nucleic acid binding	Down	YT-M, YT-E
DF	GO:0003723	RNA binding	Down	YT-M, YT-E
DF	GO:0003735	Structural constituent of ribosome	Down	YT-M, YT-E
DF	GO:0005506	Iron ion binding	Down	YT-M, YT-E
DF	GO:0006260	DNA replication	Down	YT-M, YT-E, LB
DF	GO:0006281	DNA repair	Down	YT-M
DF	GO:0006412	Translation	Down	YT-M, YT-E
DF	GO:0006457	Protein folding	Down	YT-M, YT-E, LB
DF	GO:0007049	Cell cycle	Down	YT-M, YT-E, LB
DF	GO:0019843	rRNA binding	Down	YT-M, YT-E
DF	GO:0051301	Cell division	Down	YT-M, LB
DF	GO:0043565	Sequence-specific DNA binding	Down	YT-E
DF	GO:0003700	DNA binding transcription factor activity	Down	YT-E
DF	GO:0016301	Kinase activity	Up	YT-M
DF	GO:0016491	Oxidoreductase activity	Up	YT-M, YT-E, LB
DF	GO:0050660	Flavin adenine dinucleotide binding	Up	YT-M
DF	GO:0050661	NADP binding	Up	YT-M
DF	GO:0051287	NAD binding	Up	YT-M
LB	GO:0030170	pyridoxal phosphate binding	Up	YT-E
LB	GO:0050660	Flavin adenine dinucleotide binding	Up	YT-E
LB	GO:0006099	Tricarboxylic acid cycle	Up	YT-E, YT-M

Table represents all enrichments found with a false discovery rate <25% in pairwise comparisons. Enrichments found in more than one comparison have been combined.

### 3.2 Pyruvate production

**Figure 3.**
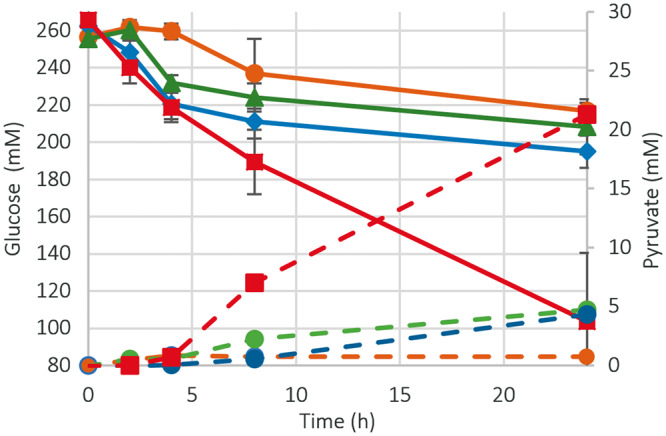
Extracts are shown here as orange, blue, green and red colored symbols and lines for DF, LB, YT-M and YT-E growth conditions, respectively. Data and standard deviation for the time course reactions were acquired using *n* = 3 biological replicates. Glucose and pyruvate were simultaneously measured at various time points over a 24-h period. Solid lines indicate glucose time courses and dashed lines indicate those for pyruvate.

We first investigated the glycolytic activity of the differentially prepared crude extracts by introducing them to a standard reaction mixture of the necessary co-factors NAD^+^ and ATP as well as a set of buffering reagents and salts in order to confirm their ability to consume glucose and drive glycolysis towards pyruvate production. Over the course of a 24-h incubation, aliquots of each reaction were halted using TCA, and quantified for glucose by HPLC analyses. Each extract broke down different amounts of glucose with YT-E consuming the largest amount at 147 mM at an average rate of 6.75 mM h^−^^1^ over a 24 h period (Figure 3).

The concentration of pyruvate was simultaneously analyzed along with glucose consumption for each of the prepared extracts over a 24-h time period. As would be expected, the final concentrations of pyruvate complemented the consumption rate of glucose with the YT-E extract producing the greatest amount of pyruvate at 21.29 mM. The DF extract produced the least amount of pyruvate at 0.73 mM and the values for LB and YT-M fell in between. However, the conversion of glucose to pyruvate was not quantitative. The differences in the extract’s ability to both consume glucose and produce pyruvate, implies that CFME extracts can have a variety of metabolic capabilities based on their different protein content that results from changes in the cell growth conditions.

**Figure 4.**
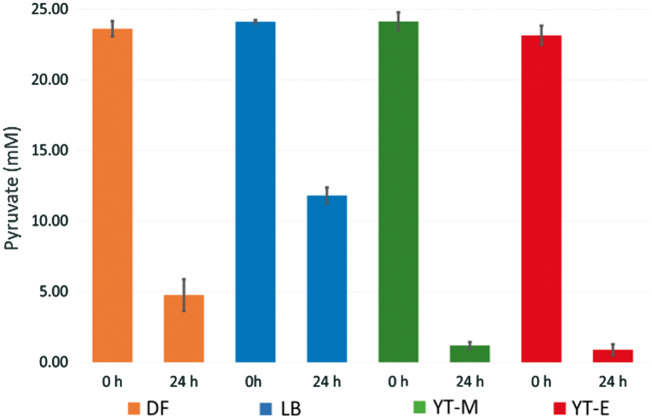
Extracts are shown here as orange, blue, green and red colored symbols and lines for DF, LB, YT-M and YT-E growth conditions, respectively. Data and standard deviation for the pyruvate substrate reactions were acquired using *n* = 3 biological replicates. The extracts’ ability to consume exogeneous pyruvate was measured. Time 0 h indicates initial pyruvate concentration. Final concentration is measured after 24 h.

Due to the breadth of potential metabolic pathways present in the crude extract, we next sought to understand if the presence of the targeted metabolite, pyruvate, was maintained at a sufficient level to be an adequate feedstock for subsequent metabolic conversion. To test the activity of the extract’s downstream pyruvate consumption pathways, we exogenously added pyruvate and co-factors to each extract and measured total pyruvate consumption after a 24-h period (Figure 4). As suggested from the proteomic analyses, sink pathways for glycolytic intermediates are well represented in the crude extracts. Calculation of the glucose to pooled pyruvate conversion rates and the pyruvate consumption rates indicate significant differences in the fraction of glucose that passes through pyruvate (Table 2). Each of the extracts was capable of consuming a large portion of the pyruvate provided regardless of the extract preparation condition. The YT-M extract was capable of consuming pyruvate at nearly the same rate as the YT-E extract, 0.96 mM h^−^^1^ and 0.93 mM h^−^^1^, respectively, despite the YT-E extract producing a larger pool of pyruvate after 24-h.

**Table 2.** Conversion amounts were determined using *n* = 3 biological replicates

Extract	Percentage of Glu consumed	Percentage of consumed glucose converted to pooled pyruvate^a^	Percentage of consumed glucose converted to pyruvate and downstream metabolism^b^
DF	15.55	0.92	24.55
LB	25.74	3.50	12.60
YT-M	18.52	4.58	28.83
YT-E	61.00	6.57	13.45

The percent glucose consumed and the percent glucose converted to pooled pyruvate were determined after 24 h of feeding the reactions 250 mM glucose and measuring the remaining glucose concentration and the amount of pyruvate produced, respectively. The percent of glucose converted to pyruvate and downstream metabolism was determined after measuring the consumption rate of 25 mM pyruvate after 24 h in the absence of glucose.

^a^Glucose conversion was calculated by measuring pooled pyruvate after 24 h and converting to glucose.

^b^The expected glucose used to make the pyruvate consumed by downstream metabolism was combined with the glucose consumed in order to produce the pooled pyruvate to account for the breakdown of pyruvate due to downstream metabolism and show the extract’s ability to synthesize glucose from pyruvate without the sink of downstream metabolism.

The DF condition was capable of breaking down pyruvate nearly 26 times faster than it could be produced indicating that the extract is relatively enriched for pathways downstream of pyruvate, in addition to those that deplete glycolytic intermediates. In the YT-M and the YT-E conditions, the consumption of pyruvate was greater than the DF condition, however, the potential consumption from downstream pathways was not enough to deplete the pyruvate reservoirs created by the extract. The YT-E extract, in particular, was able to maintain a reservoir of pyruvate that nearly matched the total pyruvate that it could consume within a 24-h time frame.

## 4. Discussion

The metabolic potential of crude extract preparations and their use for metabolite production can be assessed through exploration of their proteomic and metabolic profiles. Despite progress in the use of crude extracts for protein expression, the actual content of a crude extract and its metabolic potential is poorly understood. We sought to address this deficiency by exploring the protein profiles of different cell extract preparations and assess their ability to produce pyruvate from glucose in CFME. As a central player in a variety of cellular processes, pyruvate is not only a key indicator of a crude extract’s glycolytic potential, but also an important proxy for the extract’s ability to produce small molecules of commercial interest (21). To explore the optimization of precursor production in cell-free extracts, we modulated cell growth conditions in order to create global changes in an extract’s protein content. Given the static nature of the protein content in crude extracts, understanding the proteomic and metabolic potential of these systems can provide an effective platform onto which heterologous pathways could be engineered with predictable effects and high yields.

The ability of each crude extract to potentially break down glucose is evident from proteomic analyses. All growth conditions resulted in extracts with the presence of at least a minimal set of the ten enzymes required for converting glucose to pyruvate. Confirmation of glycolytic activity was supported by metabolite analyses. The different extracts all converted glucose to pyruvate in the presence of the appropriate cofactors. Further, a nearly 30-fold difference in the amount of pooled pyruvate, after 24-h, is observed when comparing the different crude extracts; ranging from 21.29 mM in YT-E and 0.73 mM in DF. These comparisons showcase the importance of growth conditions when preparing an extract. Proteomic analyses aid in interpreting the effect of these changes. For example, the differences in methodology to create the YT-E and the YT-M extracts are seemingly minor, with the YT-E extract being harvested at an earlier point in the growth phase when compared to YT-M. However, the effects of this change significantly impact the metabolic pathways present in the extract. While both the YT-M and the YT-E extracts are able to break down pyruvate at a similar rate, the production of pooled pyruvate in the YT-M extract is only one-fifth that of the YT-E extracts. This difference can be attributed to the prevalence of the glucose-specific PTS system, which may serve as both an entry and exit for glycolysis and account for both the increased glucose consumption and the increased production of pyruvate in the YT-E extract. The absence of the HPr protein in the DF condition removes this alternative glucose consumption pathway despite a relative abundance of the glucose-specific proteins.

Glucose consumption in cell-free systems can be a robust process and has been shown in previous studies (13). As confirmed here, 15–60% of added glucose can be metabolized by crude extract preparations. Biosynthesis and degradation pathways drawing from central metabolism, such as those for nucleotides, lipids and amino acids, can affect the flow of carbon to pyruvate in the DF cell extract. Proteome analyses indicated that the DF cell extract, which was grown on a minimal medium, is enriched in amino acid and nucleotide biosynthesis pathways that are not prevalent in the other extract preparations (Figure 2, Supplementary Table S3). These pathways rely upon intermediates from glycolysis for their carbon backbones and decrease overall flux towards pyruvate. The upregulation of the glycolytic enzymes combined with the presence of shunting pathways show a clear path by which the DF extract could produce pyruvate, but not accumulate pyruvate, in the same fashion as the YT-E extract where the overwhelming amount of the produced pyruvate was shunted downstream.

Each tested growth condition resulted in an extract capable of breaking down pyruvate, which depletes the pool of this metabolite. The different crude extracts were capable of consuming up to 90% of added pyruvate. After accounting for this consumption, the overall production rates of pyruvate for the DF, LB, YT-M and YT-E extracts are 0.82 mM h^−^^1^, 0.71 mM h^−^^1^, 1.14 mM h^−^^1^ and 1.82 mM h^−^^1^, respectively. None of the consumed pyruvate appears to be converted back to glucose. As previously noted, the production of pyruvate from PEP is effectively irreversible (28). The pyruvate is likely funneled into downstream metabolic pathways, and analyses of proteomic information provided insights. GSEA reveals that carbohydrate metabolism, specifically the Krebs cycle was up-regulated in the LB extract. Conversely, the YT-E and YT-M extracts were relatively depleted in the Krebs cycle as is common for cells in exponential growth (29). Interestingly, a component of the pyruvate dehydrogenase complex (2.3.1.12) was upregulated in both the YT-E and YT-M extracts, potentially indicating the channeling of pyruvate to acetyl-CoA. Rapid growth, which might be expected under conditions with abundant resources, results in a need for biomass components, the biosynthesis of which both starts from intermediates in glycolysis, and heavily deplete the central precursors therein (30). Extracts derived from rapidly reproducing cells can result in active biomass accumulation pathways and can result in a significant drain on both feed metabolites and cofactors in metabolic engineering endeavors. These pathways likely lead to the increased consumption of pyruvate observed in the YT-E and YT-M extracts. While sink pathways draw carbon away from central metabolism, their effect may be mitigated by the prevalence of upstream pathways providing a balancing effect. The LB extract is upregulated in both anaplerotic pathways and gluconeogenesis (4.1.3.1, 4.1.1.49, 3.1.3.11) and consumed comparatively less pyruvate than the other extracts.

The use of proteomic and biochemical analyses to describe the metabolic capabilities of a crude extract provides a useful framework for realizing an extract’s potential applications and optimization. Changes, either genetic or to growth conditions, can be made to further tailor a crude extract for desired function. Here, it is evident that growth on a minimal medium results in the expression of many sink pathways for glycolytic intermediates. Moreover, it appears specific growth rate, which has been previously examined as a key variable in CFPS extract preparation, plays a role in reducing sinks due to the Krebs cycle, but at the price of directing flux towards undetermined biomass accumulation pathways (23). Proteomic analysis is a robust technique for determining candidates for genetic manipulation and can guide *in vivo* protein overexpression or knockdowns in source strains that will affect the flux of small molecules after extract preparation. Alternative strategies, such as targeted protein degradation and pull downs, have been described for the removal of deleterious proteins from crude extracts after cell lysis to avoid negatively impacting cell growth and survival (31). Crude extracts made from high-yielding *in vivo* pyruvate production strains represent another opportunity to use *in vitro* synthetic biology to enable metabolic engineering (32,33). Some of the highest producing strains are limited in their genetic tractability, but omics data can provide strong candidates for modification and minimize the amount of genetic engineering needed.

## 5. Conclusion

Critically analyzing the central precursors of cell-free systems as well as how the conditions in which these extracts are grown can impact the metabolic potential of a cell free system due to changes in the underlying proteomic content. Notably, we demonstrate that simple changes in cell-free extract preparation can result in profound differences in metabolite pooling. Further, these changes in extract preparation have the potential to deplete important precursors that could be used for synthesis of a final product. These different metabolic characteristics can be interpreted through the combined use proteomics and metabolomics techniques. These analytical measurements further our understanding of the composition of cell-free extracts and provide a rich dataset from which to engineer improved solutions for metabolite production. These tools can guide genetic manipulations and strain optimization conditions for maximizing the production of pyruvate, as well as other important biosynthetic precursors. Feasibly, effective development of crude extracts can lead to a general platform suitable for testing biochemical pathways and for production of useful metabolites.

## SUPPLEMENTARY DATA

Supplementary Data are available at SYNBIO Online.

## Funding

This article has been authored by UT-Battelle, LLC under Contract No. DE-AC05-00OR22725 with the U.S. Department of Energy. This work was supported by the U.S. Department of Energy (DOE) Office of Biological and Environmental Research, Genomic Science Program and also in part by an appointment to the Higher Education Research Experiences Program at Oak Ridge National Laboratory for JTD. Oak Ridge National Laboratory is managed by UT-Battelle, LLC, for the U.S. DOE under Contract no. DEAC05-00OR22725.

*Conflict of interest statement.* None declared.

## Supplementary Material

Supplementary Data

Supplementary Table S1

Supplementary Table S2

Supplementary Table S3
