# Supplementary material for: Elucidating the potential of crude cell extracts for producing pyruvate from glucose
Source: Synth Biol (Oxf). 2018 May 14;3(1):ysy006. doi: 10.1093/synbio/ysy006 (PMC7445776; doi:10.1093/synbio/ysy006)
Supplement: Supplementary Data [file ysy006_supp.docx]

**Elucidating the potential of crude cell extracts for producing pyruvate from glucose**

**David C. Garcia^a,b,1^, Benjamin P. Mohr^a,b,1^, Jakob T. Dovgan^b^, Gregory B. Hurst^c^, Robert F.** **Standaert^b^, Mitchel J. Doktycz^a,b,*^
^a^Bredesen Center for Interdisciplinary Research, University of Tennessee, Knoxville, TN, USA; ^b^ Biosciences and ^c^ Chemical Sciences Divisions, Oak Ridge National Laboratory, Oak Ridge, TN, USA;

^1^ Both Authors Contributed Equally to this Work
*Corresponding Author: M. J. Doktycz: doktyczmj@ornl.gov

Supporting Information**

**Table of Contents**

Legend for Supplemental Table S1 S-2

Legend for Supplemental Table S2 S-3

Legend for Supplemental Table S3 S-3

Supplemental References S-4

**Supplemental Table S1** (prot_supp_table.xlsx) includes a complete listing of the 1,763 proteins (one per row) identified by full proteome characterizations of crude cell extracts prepared from four different growth conditions. Columns in this table are:

**Protein**: protein identifier from FASTA file used for Myrimatch searches (see Methods: 2.4 Proteomics)

A set of 7 columns listing quantities measured or calculated from each LC-MS/MS measurement; “NA” indicates that a protein was not identified in that LC-MS/MS analysis:

**SqC**: Sequence Count, the number of identified tryptic peptides for the protein, with different charge states of the same peptide counting as 1 sequence. Each identified modified form of a peptide is counted as a separate sequence.

**uSqC**: number of identified sequences that are unique within the FASTA file to this protein

**SpC**: Spectrum Count, the number of identified tandem mass spectra for this protein

**uSpC**: Spectrum Count of peptides that are unique within the FASTA file to this protein

**dSpC**: Distributed Spectrum Count, modified to account for peptides shared with other proteins (Zhang et al., 2010)

**PercentCoverage**: fraction of protein sequence covered by detected tryptic peptides

**NSAF**: Normalized Spectral Abundance Factor: corrected and normalized estimate of protein abundance. NSAF sums to 1 across all proteins in a single run (Zybailov et al., 2006)

Headers for each of these 7 columns include the sample identifier:

YT-E 1, YT-E 2, YT-E 3: LC-MS/MS biological replicates of standard *Escherichia coli* cell-free protein synthesis crude extract. Extract was grown on 2xYTPG media and collected during early-log phase.

YT-M 1, YT-M 2, YT-M 3: LC-MS/MS biological replicates of *E. coli* crude extract. Extract was grown on 2xYTPG media and collected during mid-log phase.

DF 1, DF 2, DF 3: LC-MS/MS biological replicates of *E. coli* crude extract. Extract was grown on M9 minimal media with fructose and collected during mid-log phase.

LB 1, LB 2, LB 3: LC-MS/MS biological replicates of *E. coli* crude extract. Extract was grown on M9 minimal media with fructose and collected during mid-log phase.

**other**_**prots**: Proteins in the FASTA file that share identified tryptic peptide sequence(s) with this protein Length: number of amino acids in the protein

**Description**: protein description from identifier line of the protein FASTA file

**KO**: K number orthology term assigned by BLASTKOALA (Kanehisa et al., 2016)

**Score**: weighted sum BLAST bit scores for each K number group

**Supplemental Table S2** (pep_supp.xlsx) lists tryptic peptides identified in full proteome characterizations of crude cell extracts prepared from four different growth conditions. Columns in this table are:

**Sequence**: amino acid sequence of the peptide. A number in square brackets indicates a modification in mass to the preceding amino acid.

**Z**: charge state of the peptide.

A set of 3 columns listing quantities measured or calculated from each LC-MS/MS measurement; “NA” indicates that a peptide was not identified in that LC-MS/MS analysis:

**Q**: maximum Q value (IDPicker false discovery estimate for peptide-spectrum matches) among identifications of this peptide (peptide-spectrum matches) that pass filters documented in Methods: Proteomics data analysis.

**Precursor m/z**: mass-to-charge ratio of the precursor ion that was fragmented, averaged across the spectra identified for this peptide

**Spectra**: Number of tandem mass spectra matched with this peptide sequence by Myrimatch

Headers for each of these 3 columns include a sample identifier, listed above for Supplemental Table 1.

**Proteins**: Proteins that contain this amino acid sequence

**Supplemental Table S3** (glycolytic_sink_statistics.xlsx) lists each enzyme in *E. coli* proteome UP000002032, as both an EC number and proteome accession number, that consumes or produces an intermediate of glycolysis. Columns in this table are:

**Glycolytic intermediate and Enzyme Commission number**: lists the intermediate produced or consume and the EC number of the enzyme involved.

**Protein accession number**: the accession number for the protein in the provided proteome.

**Average NSAF**: as in **Table S1**. NSAFs were omitted for proteins that fell beneath the abundance threshold or were not detected.

**ANOVA**: Benjamini-Hochberg corrected p-values for One-way ANOVA. Values were omitted for corrected p-values > 0.05.

**Supplemental references**

Kanehisa, M., Sato, Y., and Morishima, K. (2016). BlastKOALA and GhostKOALA: KEGG Tools for Functional Characterization of Genome and Metagenome Sequences. J. Mol. Biol. *428*, 726–731.

Zhang, Y., Wen, Z., Washburn, M.P., and Florens, L. (2010). Refinements to Label Free Proteome Quantitation: How to Deal with Peptides Shared by Multiple Proteins. Anal. Chem. *82*, 2272–2281.

Zybailov, B., Mosley, A.L., Sardiu, M.E., Coleman, M.K., Florens, L., and Washburn, M.P. (2006). Statistical Analysis of Membrane Proteome Expression Changes in *Saccharomyces* *c* *erevisiae*. J. Proteome Res. *5*, 2339–2347.
